# NF-κB Inducing Kinase, NIK Mediates Cigarette Smoke/TNFα-Induced Histone Acetylation and Inflammation through Differential Activation of IKKs

**DOI:** 10.1371/journal.pone.0023488

**Published:** 2011-08-24

**Authors:** Sangwoon Chung, Isaac K. Sundar, Jae-Woong Hwang, Fiona E. Yull, Timothy S. Blackwell, Vuokko L. Kinnula, Michael Bulger, Hongwei Yao, Irfan Rahman

**Affiliations:** 1 Department of Environmental Medicine, Lung Biology and Disease Program, University of Rochester Medical Center, Rochester, New York, United States of America; 2 Division of Allergy, Pulmonary and Critical Care Medicine, Vanderbilt University School of Medicine, Nashville, Tennessee, United States of America; 3 Department of Cancer Biology, Vanderbilt University School of Medicine, Nashville, Tennessee, United States of America; 4 Pulmonary Division, Department of Medicine, University of Helsinki and Helsinki University Central Hospital, Helsinki, Finland; 5 Department of Pediatrics, University of Rochester Medical Center, Rochester, New York, United States of America; University of Bristol, United Kingdom

## Abstract

**Background:**

Nuclear factor (NF)-κB inducing kinase (NIK) is a central player in the non-canonical NF κB pathway, which phosphorylates IκB kinase α (IKKα) resulting in enhancement of target gene expression. We have recently shown that IKKα responds to a variety of stimuli including oxidants and cigarette smoke (CS) regulating the histone modification in addition to its role in NF-κB activation. However, the primary signaling mechanism linking CS-mediated oxidative stress and TNFα with histone acetylation and pro-inflammatory gene transcription is not well understood. We hypothesized that CS and TNFα increase NIK levels causing phosphorylation of IKKα, which leads to histone acetylation.

**Methodology:**

To test this hypothesis, we investigated whether NIK mediates effects of CS and TNFα on histone acetylation in human lung epithelial cells *in vitro* and in lungs of mouse exposed to CS *in vivo*. CS increased the phosphorylation levels of IKKα/NIK in lung epithelial cells and mouse lungs. NIK is accumulated in the nuclear compartment, and is recruited to the promoters of pro-inflammatory genes, to induce posttranslational acetylation of histones in response to CS and TNFα. Cells in which NIK is knocked down using siRNA showed partial attenuation of CSE- and TNFα-induced acetylation of histone H3 on pro-inflammatory gene promoters. Additional study to determine the role of IKKβ/NF-κB pathway in CS-induced histone acetylation suggests that the canonical pathway does not play a role in histone acetylation particularly in response to CS in mouse lungs.

**Conclusions:**

Overall, our findings provide a novel role for NIK in CS- and TNFα-induced histone acetylation, especially on histone H3K9.

## Introduction

Cigarette smoke (CS) contains numerous reactive oxygen/nitrogen species, reactive aldehydes, and quinones [1], which are involved in the pathogenesis of chronic inflammatory lung diseases, such as chronic obstructive pulmonary disease (COPD) and lung cancer [2], [3], [4], [5]. The airway epithelium is susceptible to abnormal inflammatory response due to direct exposure of exogenous inhaled oxidants, such as tobacco smoke, airborne particulate matters, and air pollutants. Monocytes/macrophages are important cells in the pathogenesis of COPD due to the release of reactive oxygen species, lipid mediators, cytokines, chemokines, and matrix metalloproteinases [6], [7], [8]. Studies from our laboratory have shown that CS induced the inflammatory response in macrophages [9], [10] and airway epithelial cells [11], [12]. Both of these cell types can influence airway inflammation leading to airway abnormalities in COPD. Inhalation of CS and airborne particulate matter resulted in lung injury by generation of oxidative stress, leading to cascades of signaling events triggering the production of cytokines, chemokines, and other factors [7], [13], [14]. Tumor necrosis factor α (TNFα) is a ubiquitous pro-inflammatory cytokine and is induced by CS-derived oxidative stress via the activation of redox sensitive transcription factors in the lungs [15], [16], [17], [18].

Histone acetylation is an epigenetic event that plays a key role in transcription of CS-induced pro-inflammatory genes in the lung [19], [20], [21]. Increased acetylation of lysine (K) residues on histone H3 (K9, K14, K27) and H4 (K5, K8, K12) results in neutralization of positive charge on histone tails and facilitates access to transcription factors [7], [22], [23]. Recently, we have shown that there is increased acetylation of histones H3 and H4 near the promoters of pro-inflammatory genes in rodent lungs in response to CS exposure, leading to heightened inflammatory response [9], [10], [24]. Despite strong evidence of a link between CS and inflammation, the signaling mechanism by which CS and TNFα mediate pro-inflammatory effects by histone acetylation is not well understood.

The canonical nuclear factor (NF)-κB signaling pathway requires IKKβ activation for IκBα degradation. However, NF-κB inducing kinase (NIK) is a central player in the non-canonical pathway of NF-κB activation. IKKα, but not IKKβ, is a preferential substrate for NIK, as inhibition of IKKα blocks NIK-induced phosphorylation of IKKβ [25]. In a reciprocal study, inactive mutants of IKKβ do not block NIK-mediated IKKα activation [25], [26]. A recent study has demonstrated the regulation of IKKβ in TNFα-induced IKKα activation in mouse embryonic fibroblast cells [27]. Thus, NIK may impact both canonical and non-canonical NF-κB pathways *via* phosphorylation of IKK complex. However, the upstream regulatory role of NIK in modulation of IKKα-mediated histone acetylation by CS and a pro-inflammatory agent, TNFα, is not yet understood. We hypothesized that CS and TNFα increase NIK protein levels resulting in increased phosphorylation of IKKα and leading to histone acetylation. We determined the role of the NIK-IKKα pathway in phosphorylation and acetylation of histones H3 and H4 on pro-inflammatory promoters using the loss in function of NIK in human lung epithelial cells *in vitro*, and in lungs of mice exposed to CS *in vivo*.

Recently, we demonstrated that CS caused phosphorylation and activation of both IKKα and IKKβ, which was associated with NF-κB-dependent pro-inflammatory gene transcription [9], [24], [28]. Hence, we also studied the role of IKKβ-dependent NF-κB activation in CS-mediated histone acetylation using mice overexpressing IKKβ (IKTA) exposed to CS. Finally, to corroborate the *in vitro* and *in vivo* studies, we studied the activation of NIK-IKKα in resected lung samples obtained from non-smokers, smokers, and patients with COPD.

## Materials and Methods

### Ethics statement

All experimental protocols described in this study were performed in accordance with the standards established by the US Animal Welfare Acts, as set forth by the NIH guidelines, and the research protocol for these studies was approved by the University of Rochester Committee on Animal Research. The Institutional Animal Care and Use Committee affiliated as UCAR (University Committee on Animal Resources) at the University of Rochester Medical Center reviewed and approved the protocols and procedures described in this manuscript (UCAR #2007-070). The facilities and programs of the vivarium are fully accredited by the American Association of Laboratory Animal Medicine and the American Association for Accreditation of Laboratory Animal Care and are in compliance with state law, federal statute and NIH policy.

Lung tissue specimens from subjects/patients undergoing resection for suspected lung tumor or undergoing lung transplantation were collected from the Departments of Medicine and Pathology, Helsinki University Central Hospital as described in our previous study. The use of these tissues was approved by the ethics committee of the Helsinki University Central Hospital, Helsinki, Finland. Written informed consent was obtained from all study participants.

### Materials

Unless otherwise stated, all biochemical reagents used in this study were purchased from Sigma (St. Louis, MO). Antibodies against NIK (#4994), acetylated RelA/p65 (Lys310) (#3035), acetylated and phosphorylated histone H3 (Lys9/Ser10) (#9711), histone H3 (#9715), acetylated histone H4 (Lys8) (#2591), and histone H4 (#2592) were purchased from Cell Signaling Technology (Danvers, MA). Antibodies against NIK (sc-7211), NF-κB RelA/p65 (sc-372), IKKα (sc-7606 and sc-7218), and Lamin B (sc-6216) were obtained from Santa Cruz Biotechnology (Santa Cruz, CA). Anti-acetyl-histone H3 (#06-599), anti-phospho-IKKα (#07-837) and anti-IKKα (#05-536) antibodies were purchased from Millipore (Temecula, CA). Anti-phospho-IKKα (ab17943), anti-E-cadherin (ab1416), and anti-actin (CP01) antibodies were purchased from Abcam (Cambridge, MA) and Calbiochem (La Jolla, CA), respectively.

### Cell Culture

Human bronchial epithelial (H292) cells were purchased from the American Type Tissue Culture Collection (Manassas, VA). H292 cells were grown in RPMI-1640 medium (Invitrogen, Carlsbad, CA) supplemented with 10% FBS, 2 mM L-glutamine, 100 µg/ml penicillin, and 100 U/ml streptomycin. Primary normal human bronchial/tracheal epithelial (NHBE) cells, as well as their growth media (BEGM, basal epithelial growth medium), were obtained from Lonza (Walkersville, MD). NHBE cells were maintained in serum-free BEGM®, supplemented with bovine pituitary extract, hEGF, hydrocortisone, epinephrine, transferring, insulin, and retinoic acid. Since NHBE cells become irreversibly contact-inhibited, they were subcultured or used when they reached 80% confluence or less. The cells were then cultured at 37°C in a humidified atmosphere containing 5% CO_2_.

### Preparation of aqueous cigarette smoke extract

The preparation of aqueous cigarette smoke extract (CSE) is described previously in detail [10], [11], [12]. Freshly prepared CSE was diluted with culture medium containing 1% FBS, 2 mM L-glutamine, 100 µg/ml penicillin, and 100 U/ml streptomycin immediately before use for each experiment. Control medium was prepared by bubbling air through 10 ml of culture medium supplemented with 1% FBS, 2 mM L-glutamine, 100 µg/ml penicillin, and 100 U/ml streptomycin adjusting pH to 7.4, and sterile filtered as described for CSE preparation.

### Transfection

The dominant negative (DN)-NIK plasmid with NIK mutant domain on K429 and K430 (K429/430A) was obtained as described previously [29], [30]. Mock-transfected cells were used as control. The NIK small interfering RNA (siRNA, Dharmacon, Lafayette, CO) was used to knockdown NIK. Transient transfection was performed with 1–4 µg of plasmids, scrambled control siRNA, and 10–100 nM of siRNA in the presence of Lipofectamine 2000 transfection reagent (Invitrogen) in H292 cells. The transfection efficiency in case of plasmid transfection was more than 80%. One day after transfection, H292 cells were treated with CSE. Whole cell lysate and acid histone extracts were used for immunoblot analysis as described below.

### Transgenic IKTA mice

IKTA (for cIKKβ Trans-Activated) mice on a background of C57BL/6J were kindly provided by Dr. Timothy Blackwell (Vanderbilt University School of Medicine, Nashville, TN) [31]. IKTA mice express a FLAG-tagged activated form of human IKKβ (NF-κB activator) under control of the tet-O_7_ enhancer/promoter were used [31], [32]. These mice, which conditionally induce the canonical NF-κB pathway exclusively in the airway epithelium, enable studies of lung inflammatory responses. Transgene expression in airway epithelium was induced by the addition of 2 mg/ml doxycycline (Dox) in 2% sucrose to drinking water for 2 days prior to CS exposure. The water bottle was covered in aluminum foil to prevent light-induced Dox degradation. Adult wild-type (WT) C57BL/6J mice (8–12 weeks of ages) were purchased from the Jackson Laboratory (Bar Harbor, ME). IKTA and WT mice were bred and maintained under specific pathogen-free condition in the Vivarium Facility of the University of Rochester. All experimental protocols were performed in accordance with the standards established by the US Animal Welfare Acts, as set forth by the NIH guidelines and the research protocol for these studies was approved by the University of Rochester Committee on Animal Research (UCAR #2007-070).

### Mouse cigarette smoke exposure

Acute CS exposure (3 days) in WT and IKTA mice were performed as described previously [24]. In brief, mice were placed in an individual compartment of a wire cage, which was placed inside a closed plastic box connected to the smoke source. Research grade cigarettes [2R4F (total particulate matter per cubic meter of air (TPM) concentration 11.7 mg/cigarette, tar 9.7 mg/cigarette, nicotine 0.85 mg/cigarette), University of Kentucky, Lexington, KY] were used to generate smoke and mice received two 1 hr exposures per day, 1 hr apart, according to the Federal Trade Commission protocol (1 puff/min of 2 sec duration and 35 ml volume) for 3 days using a Baumgartner-Jaeger CSM2072i automatic cigarette smoking machine (CH Technologies, Westwood, NJ). Mainstream CS was diluted with filtered air and directed into the exposure chamber. The smoke exposure was monitored in real-time with a MicroDust pro-aerosol monitor (Casella CEL, Bedford, UK) and verified daily by gravimetric sampling. The smoke concentration was set at a value of ∼300 mg/m^3^ total particulate matter (TPM) (corresponding to human consumption of 1–1.5 packs per day) by adjusting the flow rate of the diluted medical air. The control mice were exposed to filtered air in an identical chamber according to the same protocol described for CS exposure. Carbon monoxide concentration in the chamber was 290–300 ppm. The dosimetry of carbon monoxide in CS was estimated by measuring blood carboxyhemoglobin levels. Mice tolerated CS without evidence of toxicity (carboxyhemoglobin levels ∼17%, and no body weight loss) [33], [34]. Mice were anesthetized by an intraperitoneal injection of pentobarbital sodium (100 mg/kg: Abbott Laboratories, Abbott Park, IL) and then sacrificed. The lungs were removed *en bloc* and frozen for immunoblot analysis.

### Bronchoalveolar lavage (BAL)

Mice were anesthetized at 24 h after the last exposure by an intraperitoneal injection of pentobarbital sodium (100 mg/kg; Abbott Laboratories, Abbott Park, IL) followed by exsanguination. The lungs were lavaged three times with 0.7 ml of saline via a cannula inserted into the trachea. The aliquots were combined and centrifuged, and the BAL inflammatory cell pellet was resuspended in saline. The cells were stained with trypan blue (Invitrogen) and the total cell number was counted using a hemocytometer. Cytospin slides (Thermo Shandon, Pittsburgh, PA) were prepared using 50,000 cells per slide, and differential cell counts (∼500 cells/slide) were performed on cytospin-prepared slides stained with Diff-Quik (Dade Behring, Newark, DE).

### Human lung tissues

Lung tissue specimens from 37 subjects/patients including 10 life-long non-smokers, 10 current smokers with normal lung function, and 9 patients with COPD (3 former and 6 current smokers; 2 patients had been prescribed inhaled steroids) undergoing resection for suspected lung tumor (either malignant or nonmalignant-local carcinoma or hamartoma), and 8 patients (former smoker; all had been prescribed inhaled and/or low-dosage oral corticosteroids) with severe COPD undergoing lung transplantation were collected from the Departments of Medicine and Pathology, Helsinki University Central Hospital as described in our previous study [35]. The use of these tissues was approved by the ethics committee of the Helsinki University Central Hospital, Helsinki, Finland. Written informed consent was obtained from all study participants. COPD was defined according to the GOLD (Global Initiative for Chronic Obstructive Lung Disease) criteria (FEV_1_<80% of predicted, FEV_1_/FVC<70% and bronchodilatation effect <12%). None of the patients had suffered from acute exacerbation for 2 months. Tumor-free peripheral lung tissues were immediately stored at −80°C, and were used for immunoblot analysis and embedded in paraffin for immunohistochemistry. The clinical characteristics of the patients used are described in detail previously [35].

### Immunohistochemistry

Paraffin embedded lung sections (3 µm thick) from non-smokers, smokers, and patients with COPD were de-waxed, endogenous peroxidase activity was blocked (3% H_2_O_2_, 30 min) and non-specific background prevented by blocking in 10% normal goat serum (Invitrogen) for 1 hr. For detection of NIK and p-IKKα proteins, the slides were incubated with mouse monoclonal anti-NIK and anti-p-IKKα (1∶100 dilution) at 4°C overnight in a humidified chamber. For detection of IKKα protein, the slides were incubated with rabbit polyclonal anti-IKKα (1∶100 dilution) at 4°C overnight in a humidified chamber. For detection of acetyl histone H3K9, the slides were incubated with rabbit polyclonal anti-acetyl histone H3K9 (1∶100 dilution) and mouse monoclonal E-cadherin (1∶100 dilution) at 4°C overnight in a humidified chamber. Subsequent incubations were carried out with FITC conjugated anti-mouse and Alexa Fluor 594 anti-rabbit secondary antibodies for 1 hr in dark, and then the slides were rinsed in PBS and mounted with anti-fade DAPI fluoromount (Southern Biotech, Birmingham, AL), and viewed under the Nikon TE2000-E microscope (Nikon, Tokyo, Japan).

### Immunocytochemisty

H292 cells (1×10^5^ cells/well) were fixed in 4% paraformaldehyde for 10 min. The cells were then permeabilized for 10 min in 0.3% Triton X-100 in PBS, and blocked for 1 hr using 10% normal goat serum. Samples were incubated with antibodies specific for Ac/p-histone H3 (K9/S10) and NIK (1∶100) using in a humidified chamber overnight. FITC conjugated anti-mouse and Alexa Fluor 594 anti-rabbit secondary antibodies for 1 hr in dark, and then the slides were rinsed in PBS and mounted with anti-fade DAPI fluoromount (Southern Biotech), and viewed under the Nikon TE2000-E microscope (Nikon, Tokyo, Japan).

### Immunoblot analysis

The preparation of whole cell lysate, cytoplasmic, nuclear fractions, and histone extracts from lung tissues and cells are described previously [9], [10], [24]. Protein levels in samples were measured using a BCA kit (Pierce, Rockford, IL). Lysates were separated 6.5–14% SDS-polyacrylamide gel, transferred onto nitrocellulose membranes, probed with the specific primary antibody (1∶1,000 dilution in PBS containing 0.1% Tween 20), and developed by enhanced chemiluminescence. Equivalent loading of the gel was determined by quantitation of protein as well as by reprobing membranes for actin, lamin B, histone H3, or histone H4.

### Chromatin immunoprecipitation

Chromatin immunoprecipitation (ChIP) assay was performed with Imprint ChIP kit (Sigma). H292 cells (1×10^6^ cells/well) were cross-linked with 1% formaldehyde (Pierce) for 15 min, and the cell nuclei were isolated, incubated with cell lysis buffer, and sonicated with 7×7 sec pulses. Equal amounts of sonicated chromatin were immunoprecipitated with 1 µg of each of the following antibodies: mouse IgG, acetylated histone H3 (#06-599, Millipore), and NIK. The chromatin was washed, and the crosslinks were hydrolyzed. The DNA was then purified through DNA binding column and subjected to RT-PCR analysis. Samples of input DNA were also prepared in the same way as described above. PCR amplification was performed using a PTC-200 DNA engine (M. J. Research, Waltham, MA) under the following conditions: 94°C for 3 min; 32 cycles at 94°C for 45 sec, 60°C for 1 min, and 72°C for 1 min; and final elongation at 72°C for 10 min. PCR for the input reaction was performed using 100 ng of genomic DNA. The following primer was used in PCR: *IL-6*, 5′-TTG CGA TGC TAA AGG ACG-3′ (sense) and 5′- TGT GGA GAA GGA GTT CAT AGC-3′ (antisense); *IL-8*, 5′-GTT GTA GTA TGC CCC TAA GAG-3′ (sense) and 5′-CTC AGG GCA AAC CTG AGT CAT C-3′ (antisense); *Cox-2*, 5′-CAA GGC GAT CAG TCC AGA AC-3′ (sense) and 5′-GGT AGG CTT TGC TGT CTG AG-3′ (antisense), and PCR products were analyzed on a 1.8% agarose gel.

### Statistical analysis

Results are expressed as means ± SEM. Statistical analysis of significance was calculated by one-way ANOVA followed by Fisher's protected least significant difference *post hoc* test for multigroup comparisons (StatView 5.0; SAS Institute, Cary, NC). Statistical significance is indicated in figure legends.

## Results

### CS and TNFα activate NIK-IKKα non-canonical pathway

**Figure 1:**
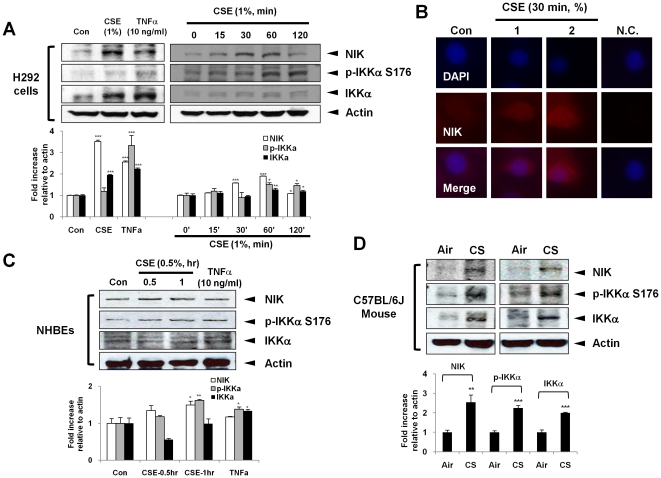
Cigarette smoke (CS) and TNFα increase the levels of NIK and p-IKKα. (A) CSE and TNFα increased the levels of NIK and phosphorylation of IKKα in H292 cells. H292 cells were treated with CSE (1%) for 1 hr or indicated time points. Whole cell extracts (30 µg) were loaded per well on SDS-6.5% polyacrylamide gel, transferred onto nitrocellulose membranes and probed with NIK, p-IKKα and IKKα antibodies. The levels of NIK and p-IKKα are significantly increased in response to CSE in a time-dependent manner. (B) For immunocytochemistry, cells were fixed, and the expression of NIK was determined by immunofluorescence. NIK is shown in red and nuclear DNA (DAPI nuclear staining) in blue. The images were taken by magnification (×400), and selected a representative cellular morphology from three separate experiments. The group without primary antibody was used as a negative control (N.C.). (C) CSE and TNFα increased the levels of NIK and phosphorylation of IKKα in NHBE cells. NHBEs were treated with CSE (0.5%) for indicated time points. (D) The levels of NIK, p-IKKα, and IKKα were increased in CS-exposed mouse lung. Gel pictures shown are representative of at least three separate experiments. After densitometry analysis, the values were normalized against the loading control, actin. Data shown as mean ± S.E. (n = 3–6 per group). *, p<0.05; **, p<0.01; ***, p<0.001, significant compared with control or air-exposed group.

We determined whether the abundance of NIK was increased by CSE and TNFα in H292 and NHBE cells. Using both immunoblot and immunofluorescent techniques, the levels of NIK were increased in response to CSE (1%) and TNFα (10 ng/ml) in H292 cells. CSE (1%) time-dependently increased NIK levels up to 60 min (Fig. 1A), which was confirmed by immunocytochemistry (Fig. 1B). The phosphorylation of IKKα at S176, which is required for activation of IKKα, was also increased after 15 min of CSE treatment in H292 cells. Treatment of NHBE cells with CSE (0.5%) resulted in a time-dependent increase in NIK and p-IKKα levels in whole cell extracts (Fig. 1C). Furthermore, the levels of NIK and p-IKKα *in vivo* in lungs of mice exposed to CS were also markedly increased (Fig. 1D).

**Figure 2:**
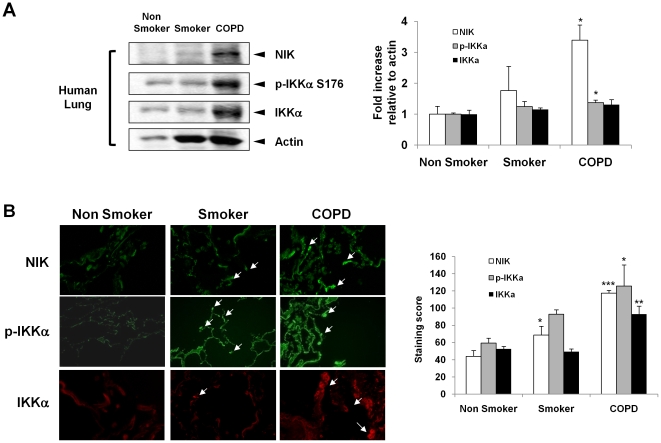
Cigarette smoke (CS) increases the levels of NIK and p-IKKα in lungs of patients with COPD. (A) Immunoblotting analysis of NIK, p-IKKα, and IKKα in lung tissue of non-smokers, smokers, and patients with COPD (n = 4–6). Gel pictures shown are representative of at least three separate experiments. After densitometry analysis, the values were normalized against the loading control, actin. (B) Representative immunofluorescent images (original magnification, ×100) showed increased levels of NIK, p-IKKα, and IKKα in lungs of smokers with and without COPD as compared to non-smokers. Arrows indicate the cells that express increased levels of NIK, p-IKKα, and IKKα. Immunostaining scores were performed semiquantitatively and in a blinded fashion. Data shown as mean ± S.E. (n = 4–6 per group). *, p<0.05; **, p<0.01; ***, p<0.001, significant compared with non-smoker group.

To further determine the levels of NIK, peripheral lung tissue samples were collected from non-smokers, smokers, and patients with COPD. The levels of NIK, p-IKKα, and IKKα were measured by immunoblotting after normalization with the amount of actin (loading control). There was a significant increase in the levels of NIK and p-IKKα in lungs of patients with COPD compared with non-smokers (Fig. 2A). The increase of NIK and p-IKKα were more pronounced in lungs of patients with COPD compared to that of smokers. Immunohistochemical staining of fixed peripheral lung tissues confirmed the increase in levels of NIK, IKKα, and p-IKKα in lungs of smokers and patients with COPD when compared with non-smokers (Fig. 2B). These results suggest that NIK/IKKα non-canonical pathway is activated by CS and may in part be responsible for increased NF-κB activation seen in lungs of smokers and patients with COPD.

### NIK is accumulated into the nucleus by CS in human lung epithelial cells and mouse lung

**Figure 3:**
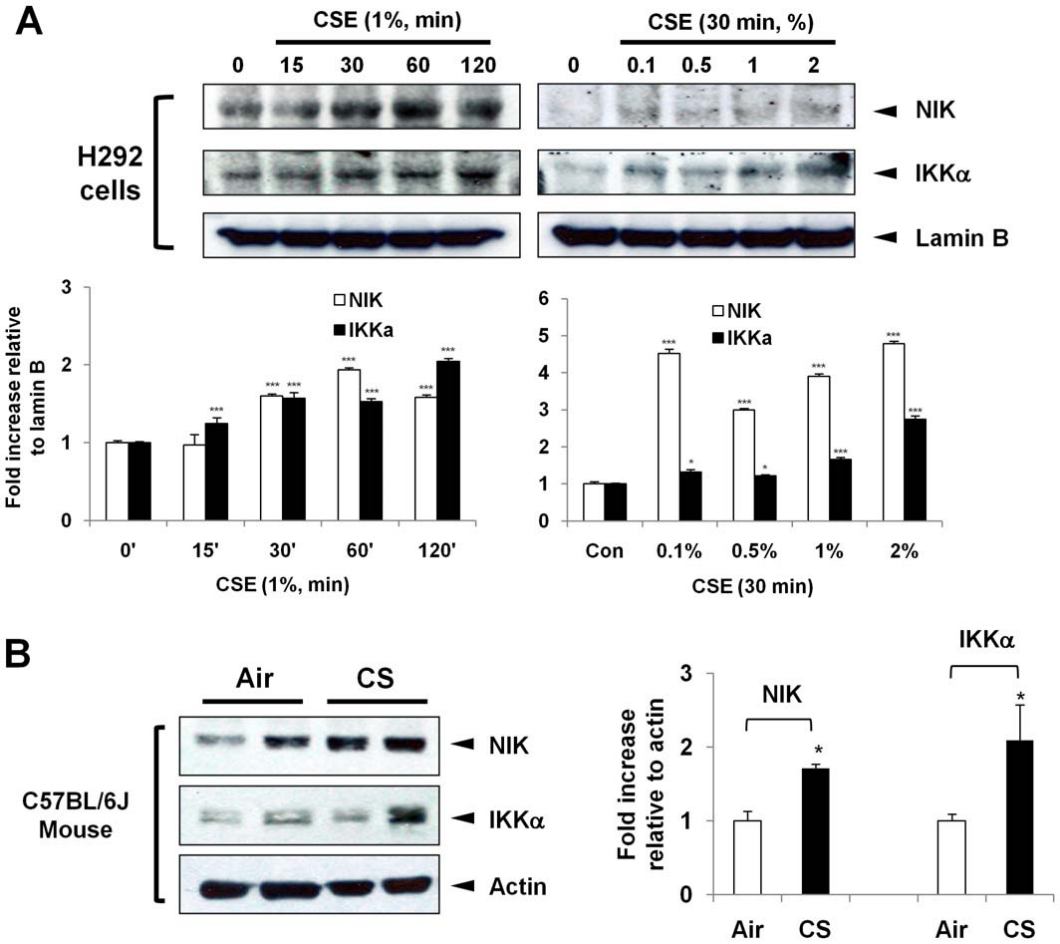
NIK and IKKα are accumulated into the nucleus in human lung epithelial cells and mouse lung in response to CS. (A) CS increased the nuclear accumulation of NIK and IKKα in H292 cells in a time- and dose-dependent manner. (B) The levels of NIK and IKKα are increased in nuclear extracts of CS-exposed mouse lung. Nuclear proteins (20 µg) were loaded in per well and performed SDS-6.5% polyacrylamide gel, transferred onto nitrocellulose membranes and probed with NIK and IKKα antibodies. Antibodies specific for nuclear LaminB demonstrate separation of the nuclear protein fractions, and equal loading. Gel pictures shown are representative of at least three separate experiments. After densitometry analysis, the values were normalized against the loading control, actin or LaminB, respectively. Data shown as mean ± S.E. (n = 3–6 per group); *, p<0.05; ***, p<0.001, significant compared with control or air-exposed group.

Nuclear accumulation and activation of NIK and IKKα are important in regulation of nuclear events [36], [37], [38]. Therefore, it is possible that CS causes the translocation of NIK and IKKα into the nucleus leading to increased transcription of pro-inflammatory mediator genes. CSE treatment resulted in nuclear accumulation of NIK and IKKα in H292 cells in a time- and dose-dependent manner (Fig. 3A). CS exposure also increased the nuclear levels of NIK and IKKα in mouse lungs (Fig. 3B). These data indicate that NIK, along with IKKα, were increased and translocated into the nucleus in response to CS in human lung epithelial cells as well as in mouse lung.

### NIK knockdown reduces CS-induced histone phosphorylation and acetylation in human lung epithelial cells

**Figure 4:**
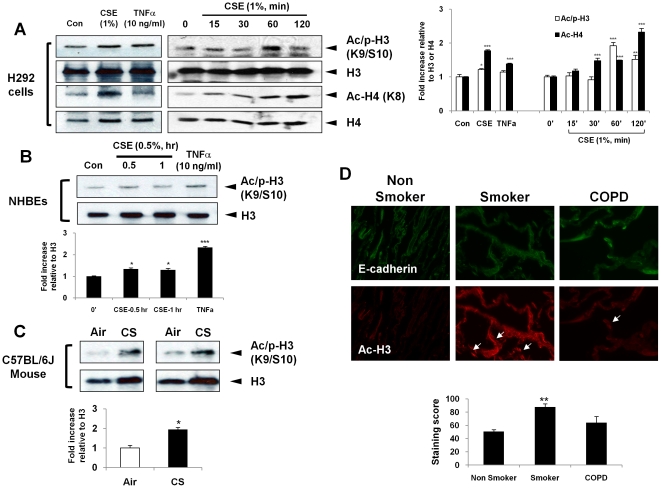
CS and TNFα mediate post-translational modification of histones. (A) Acid-extracted histone proteins were used for immunoblotting (using SDS-14% polyacrylamide gel) against phospho-acetylation of histone H3 (S10/K9) and acetylation of histone H4 (K8). CSE and TNFα increased the levels of phospho-acetylated histone H3 and acetylated histone H4 in H292 cells. H292 cells treated with CSE for indicated time points. The levels of phospho-acetylated histone H3 and acetylated histone H4 are significantly increased in response to CSE by a time-dependent manner. (B) NHBEs were treated with CSE (0.5%) and TNFα for 0.5 or 1 hr. (C) The levels of phospho-acetylated histone H3 (S10/K9) were also increased in CS-exposed mouse lung. (D) Immunofluorescence staining shows localization and increased staining of acetyl-histone H3K9 in alveolar and airway areas of lungs (shown by arrows) from smokers and in patients with COPD compared to non-smokers. E-cadherin staining was used as an epithelial marker. Immunostaining scores were performed semiquantitatively and in a blinded fashion. Gel pictures shown are representative of at least three separate experiments. After densitometry analysis, the values were normalized against the loading control, histones H3 and H4, respectively. Data shown as mean ± S.E. (n = 3–6 per group). *, p<0.05; **, p<0.01, ***, p<0.001, significant compared with control, air-exposed, or non-smoker groups, respectively.

Activation of IKKα is thought to regulate phospho-acetylation of histone H3 and acetylation of histone H4, thereby inducing chromatin remodeling [9], [38]. We found that stimuli, such as CSE and TNFα, induced histone acetylation including phospho-acetylation of histone H3 (S10/K9) and acetylation of histone H4 (K8) in both H292 (Fig. 4A) and NHBE cells (Fig. 4B). Similarly, the levels of phospho-acetylated histone H3 were increased in CS-exposed WT mouse lung (Fig. 4C). We also found that acetylation of histone H3K9 occurs in airway and alveolar epithelial cells of smokers with and without COPD (Fig. 4D).

**Figure 5:**
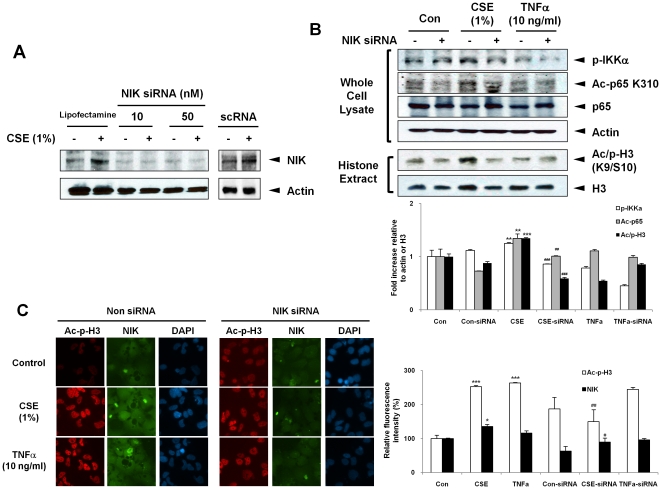
siRNA knockdown of NIK leads to reduced CSE- and TNFα-mediated post-translational modification of histones in H292 human lung epithelial cells. (A) H292 cells were transiently transfected with NIK siRNA then treated with CSE and TNFα for 1 hr. NIK expression in H292 cells treated with NIK siRNA and scrambled control siRNA (scRNA) were measured by immunoblotting. Whole cell extracts (30 µg) were loaded per well on SDS-10% polyacrylamide gel, transferred onto nitrocellulose membrane. Knockdown of NIK showed a decreased level of NIK in H292 cells in response to CSE. (B) CSE and TNFα increased post-translational modifications of IKKα, RelA/p65 (K310), and histone H3 (S10/K9), and the cells lacking NIK showed decreased levels of phosphorylated IKKα, acetylated RelA/p65, and phospho-acetylated histone H3. (C) For immunocytochemistry, phospho-acetylated histone H3 (S10/K9) is shown in red, NIK is shown in green, and nuclear DNA (DAPI nuclear staining) in blue. The fluorescence intensity values in the non-siRNA control were stated as 100% and the other values were normalized, accordingly. The images were taken by magnification (×200), shown is a representative cellular morphology from three separate experiments. After densitometry analysis, the values were normalized against the loading control, actin and histone H3, respectively. Data shown as mean ± S.E. (n = 3–6 per group). *, p<0.05; **, p<0.01, ***, p<0.001, significant compared with control group; #, p<0.05, ##, p<0.01, ###, p<0.001, significant compared with CSE-exposed group.

**Figure 6:**
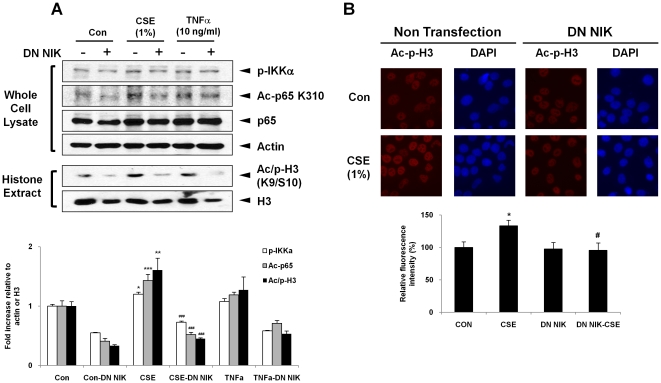
NIK dominant mutant leads to reduced CSE- and TNFα-mediated post-translational modification of histones in H292 cells. (A) H292 cells were transiently transfected with NIK dominant negative plasmid (DN NIK) and then treated with CSE and TNFα for 1 hr. The NIK mutant transfected cells reduced the enhancement of phosphorylated IKKα, acetylated RelA/p65, and phospho-acetylated histone H3 in response to CSE. (B) For immunocytochemistry, phospho-acetylated histone H3 (S10/K9) is shown in red and DNA (DAPI nuclear staining) in blue. The fluorescence intensity values in the non transfection control were stated as 100% and the other values were normalized, accordingly. The images were taken by magnification (×200), shown is a representative cellular morphology from three separate experiments. After densitometry analysis, the values were normalized against the loading control, actin and histone H3, respectively. Data shown as mean ± S.E. (n = 3–6 per group). *, p<0.05; **, p<0.01, ***, p<0.001, significant compared with control group; #, p<0.05, ###, p<0.001, significant compared with CSE-exposed group.

To assess the role of NIK in CSE-induced histone phosphorylation and acetylation, we used the siRNA approach to knockdown the expression of NIK in H292 cells. Immunoblotting demonstrated that NIK siRNA effectively decreased NIK protein levels by 70–80% as compared to controls (Fig. 5A). NIK siRNA transfected cells were treated with CSE (1%) for 1 hr, thereafter histone H3K9 modifications were assessed by immunoblotting and immunocytochemistry. As expected, the phosphorylation of IKKα was decreased after NIK siRNA (Fig. 5B) and/or dominant negative mutant transfection in H292 cells in response to CSE and TNFα treatments (Fig. 6A). The levels of phospho-acetylated histone H3 (S10/K9) were increased in response to CSE and TNFα, which was significantly decreased in cells transfected with NIK siRNA employing both immunoblotting and immunocytochemistry approaches (Fig. 5B and 5C). We also found that aceylated RelA/p65, which is associated with hyper-acetylation of histone H3K9, was increased in response to CSE and TNFα, but was lowered in NIK knockdown cells (Fig. 5B). Comparable reduction in acetylated RelA/p65 and phospho-acetylated histone H3 (S10/K9) were observed in H292 cells transfected with NIK dominant negative mutants (Fig. 6). These data suggest that NIK contributes to both CS- and TNFα-induced histone acetylation associated with phospho-acetylation of both histone H3 (S10/K9) and RelA/p65 in H292 cells.

### CSE and TNFα treatments cause the recruitment of NIK on the promoters of inflammatory genes leading to histone H3K9 acetylation

**Figure 7:**
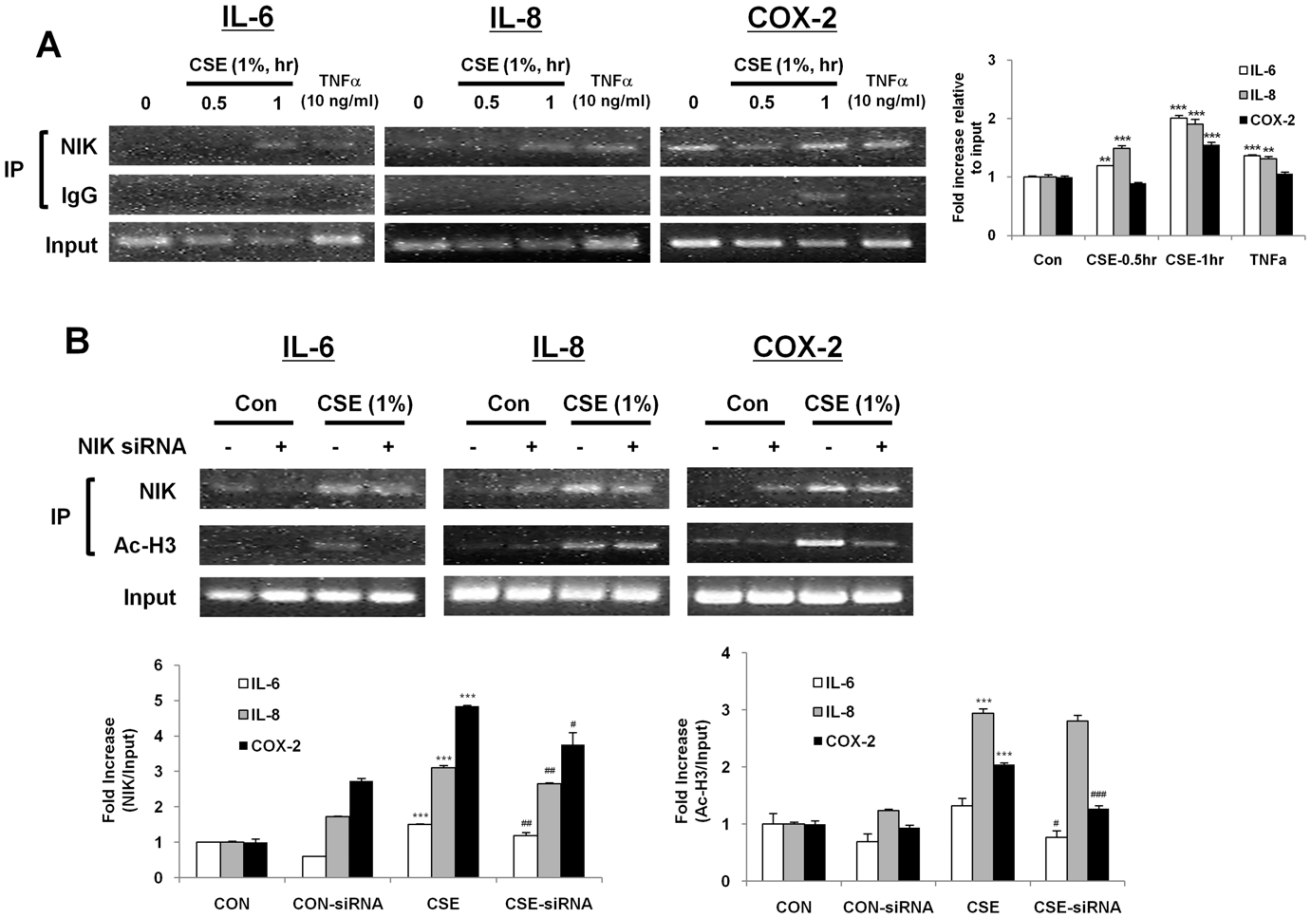
NIK is localized on the promoters of pro-inflammatory genes by CSE and TNFα leading to histone H3 acetylation in H292 cells. (A) Chromatin immunoprecipitation analysis of pro-inflammatory genes, *IL-6*, *IL-8, and COX-2* promoters with NIK antibody in H292 cells. Input DNA control is shown in the bottom panel. (B) NIK knockdown inhibited recruitment of NIK, and acetylated histone H3 to the promoters of pro-inflammatory genes in H292 cells in response to CSE. Images shown are representative of at least two separate experiments. After densitometry analysis, the values were normalized against the loading control, input. Data shown as mean ± S.E. (n = 2–3 per group). **, p<0.01, ***, p<0.001, significant compared with control group; #, p<0.05, ##, p<0.01, ###, p<0.001, significant compared with CSE-exposed group.

It has been shown that IKKα is recruited to the promoter of pro-inflammatory genes in response to CS *in vitro* epithelial cells and *in vivo* mouse lungs [9]. However, it is unclear whether its upstream kinase, NIK is also recruited on the promoters of pro-inflammatory genes. ChIP assays were performed in epithelial cell lysates using the antibody against NIK to assess its recruitment on pro-inflammatory gene promoters. It was found that increased endogenous NIK recruitment occurred on the promoters of *IL-6*, *IL-8*, and *Cox-2* in cells treated with CSE (1%) or TNFα for 1 hr (Fig. 7A). These results suggest that NIK physically localizes on chromatin associated with hyper-acetylation of histones, and thus enhances the CS-induced pro-inflammatory gene transcription.

We next performed the ChIP assays to determine the recruitment of NIK in response to CSE in NIK knockdown cells. As shown in Fig. 7B, in response to CSE, the enrichment of NIK levels on pro-inflammatory gene promoters was decreased in NIK specific siRNA transfected cells. Interestingly, the deficiency of NIK also partially attenuated the acetylation of histone H3K9 on the promoters in response to CSE (Fig. 7B). These data suggest that CS-mediated recruitment of NIK on the promoters increases the pro-inflammatory gene transcription by causing histone acetylation along with NF-κB activation.

### Canonical IKKβ/NF-κB pathway does not contribute to histone acetylation in response to CS exposure

**Figure 8:**
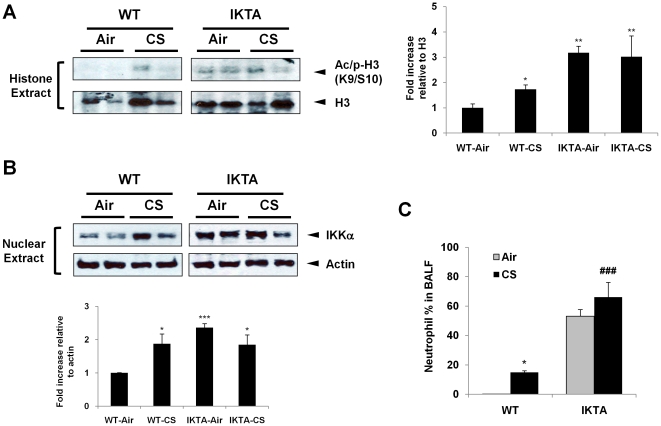
CS-induced histone phospho-acetylation is independent of the canonical NF-κB pathway. (A) Acid-extracted histone proteins from the lungs of IKTA mice were used for immunoblotting against phospho-acetylation of histone H3 (S10/K9). (B) Nuclear extracts were immunoblotted for IKKα levels from WT and IKTA mice exposed to air or CS. The levels of IKKα and phospho-acetylated histone H3 (S10/K9) were not augmented in lungs of IKTA mice as compared to WT mice exposed to CS. Gel pictures shown are representative of at least three separate experiments. (C) Neutrophil influx in BAL fluid of mice was determined on cytospin-prepared slides stained with Diff-Quik. After densitometry analysis, the values were normalized against the loading control, histone H3 and actin, respectively. Data shown as mean ± S.E. (n = 3–5 per group). *, p<0.05, **, p<0.01, ***, p<0.001, significant compared with air-exposed WT group; ###, p<0.001, significant compared with CS-exposed WT mice.

IKKα and IKKβ, the downstream of NIK, can regulate each other in response to inflammatory stimuli [25], [26], [27]. We and others have shown that IKKα plays an important role in regulating histone acetylation [9], [38]. However, the role of canonical IKKβ/NF-κB pathway in histone acetylation especially in response to CS exposure is unknown. To determine the role of IKKβ/NF-κB pathway in CS-induced histone acetylation, we utilized the strategy of transgenic mice that express constitutive IKKβ (IKTA) in airway epithelial cells after Dox induction [31]. As shown in Fig. 8A, the levels of histone H3 (S10/K9) phospho-acetylation were increased in lungs of WT mouse in response to CS. The IKTA mice showed a basal increase in the levels of phospho-acetylated histone H3 (S10/K9) compared to WT mice (Fig. 8A). To confirm and validate this result, we examined the nuclear IKKα level in IKTA mouse lungs. Similarly, there was an increase in IKKα levels without any further augmentation of histone H3 phospho-acetylation (S10/K9) in lungs of IKTA mice following CS exposure compared to WT mice (Fig. 8B). These results are consistent with the previous finding, which showed that inhibition of IKKβ leads to a compensatory increase in IKKα activation [39]. This compensatory activation of IKKα may lead to basal histone acetylation under the condition of IKKβ inhibition. Notably, IKTA mice had further enhanced neutrophil influx into BAL fluid in both air and CS exposures compared to WT mice (Fig. 8C). Taken together, these findings indicate that overexpression of IKKβ though show a compensatory activation of IKKα, does not involve in CS-induced histone acetylation in mouse lungs; albeit IKKβ/NF-κB activation is required for CS-induced neutrophil influx in the lung.

## Discussion

It is thought that CS-induced oxidative stress is the most important risk factors in the development of COPD by inducing pro-inflammatory gene transcription through chromatin modifications in the lung [10], [20], [21]. TNFα, a pro-inflammatory cytokine, can be ubiquitously generated in biological systems under the condition of oxidative stress (e.g. induced by CS) acting as a mediator of multiple inflammatory events. Recently, it has been shown that IKKα activation is critical in phospho-acetylation of histone H3 on pro-inflammatory gene promoters in response to stimuli including CS, suggesting nuclear function of IKKα [9], [24], [38]. However, the contribution of its upstream kinase, NIK in CS- and TNFα-induced histone acetylation and inflammatory responses is not known. Furthermore, it is unknown whether both the canonical and non-canonical pathways are important in histone acetylation. Herein, we hypothesized that CS and TNFα trigger the activation of NIK-IKKs complex, leading to phospho-acetylation of histones on the promoters of pro-inflammatory genes in human bronchial epithelial cells *in vitro* and in mouse lung *in vivo*. Our data revealed that CS and TNFα increased the levels of NIK and phosphorylation of IKKα at Ser176 residue in human lung epithelial cells and mouse lung in response to CS. Furthermore, IKKβ was also activated in mouse lung after CS exposure [24]. This was associated with increased phosphorylation of RelA/p65 on Ser276 and Ser536 and its acetylation on Lys310 occurred in both transformed and primary lung epithelial cells and *in vivo* in mouse lung (data not shown). Overall, these results indicate the activation of NIK-IKKs-NF-κB pathway in response to CS exposure. The mechanism underlying the activation of NIK-IKKs complex may be associated with CS-induced oxidative stress and/or a subset of tumor necrosis factor or Toll-like receptors-dependent signals [9], [40]. This is supported by previous studies of NIK, which is a redox-regulated signaling molecule in oxidative stress-mediated pro-inflammatory responses [41], [42]. It is also known that CS-mediated release of pro-inflammatory mediators act as paracrine signaling to further augment and sustain the inflammatory response in the lung [12]. However, in acute CS exposure as described in this study, the initial response by airway epithelium may be independent of initial *de novo* release of pro-inflammatory cytokines. This implicates a direct pro-inflammatory signaling effect of CS on the inflammatory transcriptional machinery.

CS is the primary cause of COPD characterized by accelerated decline in lung function and alveolar destruction of the lung [35], [43]. Over 4,000 different chemicals are present in CS, individual component are unlikely to develop COPD/emphysema compared to whole CS [1], [3], [10]. Recent research on COPD inflammation has focused on the understanding of the molecular signaling epigenetic mechanisms involved in underlying pathogenesis of lung inflammation in smokers who are susceptible to COPD. We extend this notion to peripheral human lung samples from smokers and patients with COPD. We observed the increased levels of NIK and p-IKKα in peripheral lungs of smokers and patients with COPD, a similar finding was seen in human lung epithelial cells. CSE used *in vitro* in the present study contains volatile components, including a variety of reactive aldehydes which play an important role in alteration of cell signaling and the pro-inflammatory response. For example, 10% CSE contains ∼394 µM of acrolein while smoking one cigarette yields ∼500 µg acrolein and ∼1000 µg acetaldehyde, and retention of 80–85% acrolein in the respiratory tract of animals exposed to cigarette smoke at the dose of 400–600 mg/m^3^ of total particulate matter according to the 1992 WHO/International program on Chemical Safety report. Although CSE differs from the gaseous phase of smoke, our data obtained in lung *in vivo* and lung epithelial cells *in vitro* suggest the involvement of NIK in CS-induced lung inflammatory signaling via histone acetylation. We have previously shown that CS-derived reactive components led to increased acetylation of RelA/p65 in lungs of smokers and patients with COPD [35]. Increased acetylation of RelA/p65 was associated with increased NF-κB-mediated abnormal inflammatory effect *via* pro-inflammatory gene transcription in smokers and patients with COPD. Consistent with this notion, we further found the increased acetylation of histone H3K9 in airway and alveolar epithelial cells of smokers with and without COPD. Therefore, it is possible that CS-induced activation of NIK-IKKα may in part be responsible for increased histone H3 (S10/K9) phospho-acetylation seen in lungs of smokers and patients with COPD.

CS-induced oxidative stress induces the phosphorylation and acetylation of histone H3 and H4, and it is known that chromatin modification has a key role in CS-mediated transcription of pro-inflammatory genes [9], [10], [21], [24]. The central regulatory serine/threonine kinase, NIK is possibly involved in the enhancement of target gene expressions through phosphorylation of IKKα, which in turn leads to nucleosomal modification [37], [44], [45]. It has been shown that NIK interacts with, and activates IKKα and IKKβ, however IKKα is a preferential substrate, especially at Ser176 residue [46], [47], [48]. Under normal conditions, NIK is kept in low abundance by constant degradation through the ubiquitination [40], [49]. A recent study suggests a previously uncharacterized mechanism that a negative feedback mechanism for the modulation of NIK is occurred by IKKα-mediated NIK phosphorylation, controlling the stability of NIK [45]. In contrast, aberrant degradation of NIK by certain stimuli undergoes stabilization and nuclear accumulation of NIK involved in cascades of signaling events [9], [37], [38], [50]. Nuclear NIK localization is determined by the stretch of basic amino acids in the N-terminal part of the protein [R(143)-K-K-R-K-K-K(149)] [48], [51]. The function of nuclear NIK could be related to the role of IKKα in not only phosphorylation of histone H3 (Ser10) but also RelA/p65 (Ser536), leading to facilitation of the transcriptional process [38], [52]. Our data showed that NIK is accumulated in the nuclear compartment in response to CS, though NIK undergoes constitutive degradation after 1 hr treatment with CSE in the whole cell lysate. We also observed that mutants of NIK, as well as the loss of NIK led to reduced CS or TNFα-induced phosphorylation and acetylation of RelA/p65 on Ser536 and Lys310 together with IKKα phosphorylation. These findings indicate that NIK regulates CS and/or TNFα-mediated NF-κB activation possibly by phosphorylation of IKKs complex. Additionally, we have shown that endogenous NIK is recruited on the promoter sites of pro-inflammatory genes to induce post-translational modification of histones in response to CS or TNFα. Furthermore, these modifications of histone, particularly on H3 (S10/K9), directly interacted with NIK on the promoters by CS in lung epithelial cells. Thus, in response to inflammatory cellular stimuli, NIK physically localize to chromatin to regulate pro-inflammatory gene transcription. In principle, a protein phosphorylation could facilitate activation and nuclear import. Although the exact mechanism of phospho-activation and recruitment of NIK by CS or TNFα is not known, it may be possible that CS and/or TNFα-derived reactive species/aldehydes activate upstream kinase or inhibit phosphatase leading to the sensing of NIK activation.

The physiological consequence of recruitment of NIK is observed after knockdown of NIK, that it partially attenuated CSE-induced acetylated histone H3 on *IL-6*, *IL-8*, and *Cox-2* gene promoters. These findings are in agreement with previous reports indicating that NIK has an important role in histone modifications by inducing IKKα phosphorylation as an intermediator, and might directly associate with acetylated histone H3K9 by co-activating CBP, to enhance target gene expression.

CBP is known to be recruited on promoters of NF-κB-dependent genes, and hence leading to acetylation of histone proteins. We have previously shown that CBP was recruited onto pro-inflammatory gene promoters in response to CS exposure in mouse lung [10]. Similarly, the interaction of CBP with NIK was increased in response to CS (data not shown). These data suggest that aside from non-canonical pathway of NF-κB, once NIK is activated by CSE, it forms nucleosomal structure with IKKα, CBP, and RelA/p65, which may then proceed to the acetylation of histone H3K9. However, this effect may not be global, since the binding is not reduced dramatically at all promoters in cells deficient in NIK. Besides NIK, IKKα is also recruited and interacted with histone protein in the nucleus, resulting in continuous/sustained pro-inflammatory gene transcription [9], [37], [38], [42]. This may reflect the synergistic effects, which allow both NIK and IKKα to bind to the pro-inflammatory gene promoters and drive epigenetic histone acetylation in response to cellular stress. Further studies are required to confirm this contention using the knockdown or overexpression of NIK in specific cells *in vivo*.

Histones can be methylated on lysine residues and thereby provide a recognition site for recruitment of regulatory factors [8], [53], [54]. Unlike acetylation, histone lysine methylation can signal either activation or repression, depending on the site and degree (mono-, di-, or tri-) of methylation [8]. Furthermore, distinct histone modifications can engage in “cross-talk” that affects gene regulation [55]. Therefore, analysis of histone methylation may provide useful information along with histone acetylation in inflammatory diseases including COPD, asthma, lupus, and inflammatory bowel diseases [54], [56], [57], [58], [59]. Surprisingly, we were unable to find any significant increase in histone methylation response to CSE at an early time point in H292 cells (data not shown). This might be due to the early effect of CS on histone acetylation, whereas histone methylation might ensue only at later time points. In light of this contention, Liu *et al.* have recently reported increased H3K27 trimethylation after long-term CSE exposure in bronchial epithelial cells, suggesting that CS does induce epigenomic alterations (especially histone methylation) [60]. Another possible explanation would be that CS differentially regulates a distinct pattern of histone modifications (acetylation *vs.* methylation) on pro- and anti-inflammatory gene promoters, such as IL-6 *vs.* IL-10.

The IKK complex consists of two homologous kinase subunit, IKKα and IKKβ, and regulatory subunit IKKγ [61]. IKKβ is most important for IκBα degradation leading to the classical NF-κB activation, whereas IKKα controls not only the activation of p52∶RelB dimers (referred to as the alternative pathway), but also induces histone H3 phosphorylation, which is critical for the activation of NF-κB-directed gene expression [38], [62]. Surprisingly, the nuclear role of IKKα is unaffected in IKKβ-mediated rapid NF-κB activation by pro-inflammatory signaling cascades [63], [64], [65]. In this study, the effect of modulation of the canonical pathway on CS-induced histone acetylation was also examined. Our data revealed that IKKβ overexpression especially in lung epithelium of mice contributed to basal histone acetylation which was not augmented by CS exposure in comparison with WT mice. Thus, IKKβ does not play a role in histone acetylation particularly in response to CS though there was a compensatory increase in the level of IKKα, which may be associated with histone phospho-acetylation. As expected, we observed unique differences in neutrophil influx (a surrogate marker of lung inflammation) in IKTA mice exposed to CS compared with WT mice. This suggests that the canonical pathway has effects on neutrophil influx that may be independent of the observed effects on histone acetylation by CS. Hence, both the canonical and non-canonical pathways may be involved in CS-induced lung neutrophil influx and histone modifications. Future studies are required to understand the distinct roles of IKKα *vs.* IKKβ in regulating NF-κB-dependent neutrophil influx. The indispensable function of NIK-IKKα axis in our CS model is to regulate the pro-inflammatory gene expression through histone acetylation. However, activation of NF-κB and lung neutrophil influx are dependent on IKKβ signaling in response to CS. Thus, simultaneous inhibition of IKKα and IKKβ may provide a way of synergistic targeting in resolution of CS-induced inflammatory response in the lung, and possible devising therapeutic strategies in CS-induced chronic inflammatory diseases including COPD.

**Figure 9:**
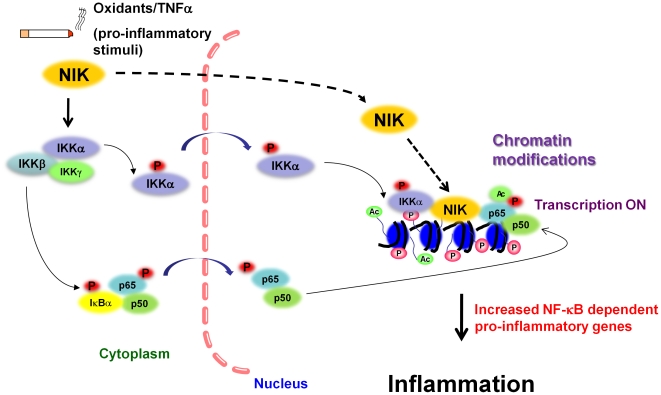
A schematic diagram for the role of NIK in CS- and TNFα-induced histone phospho-acetylation. In response to CS and pro-inflammatory stimuli, such as TNFα, NIK phosphorylates IKKα, and triggers the IKKα-mediated histone modifications. NIK is accumulated into the nucleus and is recruited on the pro-inflammatory gene promoters, resulting in increased transcription of NF-κB-dependent pro-inflammatory genes, though IKKβ is not required for this pathway. IKKβ activation leads to translocation of NF-κB dimers into the nucleus and bind to κB sites on the promoters, which is independent of NIK-IKKα activation. P, phosphorylation; Ac, acetylation.

In summary, our findings provide a novel role for NIK in CS- and TNFα-induced RelA/p65 activation and histone H3 acetylation. Enhanced accumulation of NIK into the nucleus and its recruitment onto the pro-inflammatory-gene promoters observed in human lung epithelial cells were associated with sustained NF-κB-dependent gene transcription. At the same time, these nucleosomal events, in response to CS, primarily involved in IKKα-dependent regulation do not require the activation of IKKβ-dependent canonical NF-κB pathway (Fig. 9). Thus, activation of NIK-IKKα-dependent pathway is a major contributor for histone acetylation in response to CS exposure, though both canonical and non-canonical signaling pathways can induce abnormal lung inflammation. Collectively, modulation of NIK-IKKα-dependent pathway along with IKKβ will also be a potentially key point of therapeutic target of abnormal lung inflammation. Our findings provide a new insight into the molecular mechanisms in CS-induced chromatin modifications, particularly histone acetylation and aberrant overall epigenetic pattern on pro-inflammatory promoters.
